# Mass spectrometry imaging of hair identifies daily maraviroc adherence in HPTN 069/ACTG A5305

**DOI:** 10.1371/journal.pone.0287449

**Published:** 2023-06-23

**Authors:** Elias P. Rosen, Nicole White, William M. Gilliland, Roy R. Gerona, Monica Gandhi, K. Rivet Amico, Kenneth H. Mayer, Roy M. Gulick, Angela D. M. Kashuba

**Affiliations:** 1 Eshelman School of Pharmacy, University of North Carolina at Chapel Hill at Chapel Hill, Chapel Hill, North Carolina, United States of America; 2 Department of Chemistry, Furman University, Greenville, South Carolina, United States of America; 3 Department of Obstetrics, Gynecology and Reproductive Sciences, University of California San Francisco, San Francisco, California, United States of America; 4 Department of Medicine, University of California San Francisco, San Francisco, California, United States of America; 5 School of Public Health, University of Michigan, Ann Arbor, Michigan, United States of America; 6 Fenway Health, Department of Medicine, Beth Israel Deaconess Medical Center/Harvard Medical School, Boston, Massachusetts, United States of America; 7 Weill Cornell Medicine, New York, New York, United States of America; University of Torino, ITALY

## Abstract

Objective measures of adherence for antiretrovirals used as pre-exposure prophylaxis (PrEP) are critical for improving preventative efficacy in both clinical trials and real-world application. Current objective adherence measures either reflect only recent behavior (eg days for plasma or urine) or cumulative behavior (eg months for dried blood spots). Here, we measured the accumulation of the antiretroviral drug maraviroc (MVC) in hair strands by infrared matrix-assisted laser desorption electrospray ionization (IR-MALDESI) mass spectrometry imaging (MSI) to evaluate adherence behavior longitudinally at high temporal resolution. An MSI threshold for classifying daily adherence was established using clinical samples from healthy volunteers following directly observed dosing of 1 to 7 doses MVC/week. We then used the benchmarked MSI assay to classify adherence to MVC-based PrEP regimens in hair samples collected throughout the 48-week HPTN069/ACTGA5305 study. We found that only ~32% of investigated hair samples collected during the study’s active dosing period showed consistent daily PrEP adherence throughout a retrospective period of 30 days, and also found that profiles of daily individual adherence from MSI hair analysis could identify when patients were and were not taking study drug. The assessment of adherence from MSI hair strand analysis was 62% lower than adherence classified using paired plasma samples, the latter of which may be influenced by white-coat adherence. These findings demonstrate the ability of MSI hair analysis to examine daily variability of adherence behavior over a longer-term measurement and offer the potential for longitudinal comparison with risk behavior to target patient-specific adherence interventions and improve outcomes.

## Introduction

Pre-exposure prophylaxis (PrEP) with antiretroviral drugs (ARVs) is effective against HIV-1 acquisition when sufficient drug concentrations are present during periods of exposure [1–3], conditions which rely on both PrEP adherence and persistence. Based on differences in tissue pharmacokinetics, the dosing frequency required to attain protective concentrations of ARVs can vary by route of HIV exposure [4]. As a result, PrEP guidelines for oral agents recommend daily use for most populations and event-driven dosing only for men who have sex with men. Objective measures of adherence with implementation of PrEP through clinical trials, demonstration projects, and routine clinical dissemination have shown that individual levels of adherence and persistence can be complex and often significantly lower than dosing recommendations [5–8].

Recognizing the challenge presented by daily adherence as well as the emergence of event-driven dosing, there has been an initiative to reframe an assessment of PrEP adherence in relation to dynamic risk behavior [9]. In this context, evaluating PrEP effectiveness requires information about daily changes in ARV concentrations over time which is not possible using current objective adherence measures. The period of adherence captured by pharmacologic measures varies by biological matrix based on pharmacokinetics and analysis methods. These generally fall into measurements of recent behavior (eg from plasma, saliva, or urine) or cumulative behavior (eg from intracellular metabolites in blood cells, or hair thatches) [10]. However, the picture of adherence provided by any one matrix may be different from patients’ actual daily use. Measures of recent adherence, which are susceptible to white-coat (social desirability) bias, only reflect behavior over the past few days and may not be consistent with long-term behavior. Conversely, measures of cumulative behavior provide an average adherence over a period of weeks to months, but cannot provide further differentiation of adherence patterns or how adherence coincides with risk behavior. While measures can be used in combination to couple short- and long-term adherence [11, 12], these strategies are still not capable of capturing patterns of daily adherence over an extended period of time.

Hair is a unique biomatrix for adherence assessment because each strand represents a record of systemic drug concentrations incorporated into hair from blood during follicular growth and preserved as the hair continues to grow. Sensitive analysis of hair strands by liquid chromatography-mass spectrometry (LC-MS) has been demonstrated for multiple ARV drug concentrations, which have been shown to scale proportionally with dose frequency [13] and predict virologic success [14]. LC-MS methods typically evaluate hair segments ≥ 1 cm that correspond to at least a month of growth, capturing longer-term adherence behavior but precluding an assessment of any short-term fluctuations in ARV concentrations. We have developed a new approach to measuring ARV drug exposure longitudinally along single hair strands at high spatial, and thus temporal, resolution using infrared matrix-assisted laser desorption electrospray ionization (IR-MALDESI) mass spectrometry imaging (MSI).

The HIV Prevention Trials Network (HPTN) 069/AIDS Clinical Trials Group (ACTG) A5305 study examined maraviroc (MVC) as an agent for PrEP, either alone or in combination with other antiretrovirals. Although MVC has not moved forward as a PrEP candidate, the study showed the safety and tolerability of MVC-based regimens [15, 16]. In this work, we apply IR-MALDESI MSI to: 1) characterize MVC dosing behavior through benchmarking of longitudinal MVC profiles in hair following directly observed therapy (DOT) of daily and intermittent dosing in another study called ENLIGHTEN; and, 2) investigate patterns of longer-term adherence in samples collected during the HPTN069/ACTGA5305 study, comparing these measures to commonly used adherence assessments. Using this antiretroviral as an example in our proof-of-concept study, we demonstrate the capability of MSI hair analysis to examine daily patterns of antiretroviral adherence behavior over one month via a single assay.

## Results

### IR-MALDESI MSI benchmarking of MVC in hair strands: The ENLIGHTEN study

**Fig 1:**
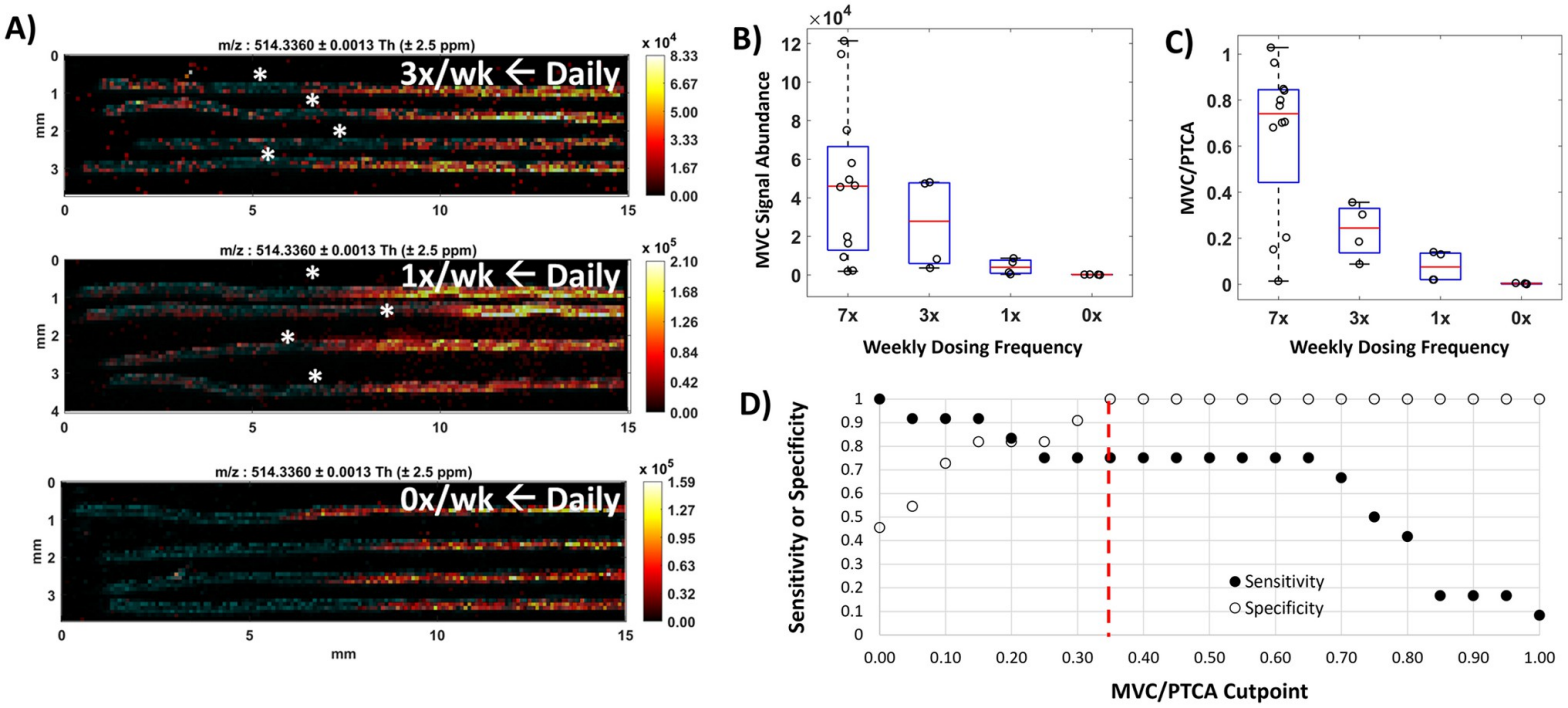
Benchmarking MVC in hair strands with MSI. (A) Representative IR-MALDESI MSI MVC ion maps showing drug accumulation associated with daily dosing and each intermittent dosing group (from top: 3x/week, 1x/week, and 0x/week, respectively) in hair strands oriented with time of growth increasing distally from left to right. MVC was measured over the proximal 15 mm, an estimated 1.5 months of growth, of samples collected at the end of the intermittent dosing period to evaluate dose-response of MVC accumulation in hair. MVC signal abundance (m/z 514.3360) is represented by a color scale increasing in concentration from regions of dark red/black to regions of orange/yellow. Cholesterol present in the hair strands (m/z 369.3516, shown in blue) is overlaid to clearly show the shape, orientation, and length of the individual strands. Apparent regions of each strand associated with a 7-day washout between dosing periods are denoted by a white asterisk. Hair was collected by clipping close to the scalp. (B) Mean IR-MALDESI MVC signal abundance associated with each ENLIGHTEN dosing group evaluated from composite longitudinal hair profiles. (C) PTCA-normalized MVC signal abundance associated with each dosing group. (D) ROC sensitivity and specificity of daily MVC adherence binary classification based on adherence cutpoint. The selected cutpoint value MVC/PTCA = 0.35 is demarcated by a red dashed line.

The ENLIGHTEN directly-observed-therapy (DOT) study provided MVC to HIV-negative volunteers in different dosing patterns. In our assessment of MVC disposition by IR-MALDESI MSI through the ENLIGHTEN study, we found that the quantitative patterns of MVC detectable along hair strands were well aligned with known dosing information, which ranged from daily (7x/week) to interrupted therapy (0, 1 or 3x/week). Regions of MVC accumulation associated with hair growth during an interrupted dosing period (3x/week, 1x/week, or 0x/week) can be seen in Fig 1A, along with higher MVC response on the right-hand, distal portion of the hair strands corresponding to growth during an earlier period of daily (7x/week) dosing. The 7-day washout interval between daily and differentiated dosing is also apparent, particularly between daily and 3x/week dosing shown at the top of Fig 1A. A time series of MSI images illustrating the movement of drug distally in hair strands collected throughout the transition between daily and intermediate dosing periods for one subject is shown in S1 Fig.

While within-individual longitudinal profiles showed MVC response scaling with dosing frequency, this observation did not hold across the whole cohort. We found that the interquartile ranges of mean MVC signal abundance from daily and intermittent dosing regions could not be differentiated (Fig 1B), indicating that raw MVC signal abundance could not reliably be used to differentiate the underlying dose frequency. Basic, lipophilic compounds like MVC can be strongly bound to melanin in hair, and we have previously observed much stronger correlation between a melanin biomarker (pyrrole-2,3,5-tricarboxylic acid, PTCA) and accumulation of MVC in hair than more hydrophilic antiretrovirals [17]. Volunteers had a range of hair colors (S1 Table) and to account for between subject variability in MVC accumulation this caused, we normalized the raw MVC signal abundance by PTCA measured in the same hair strands. Mean MVC/PTCA values can be seen in Fig 1C. Using this approach, interquartile ranges for each dosing frequency can be differentiated [MVC/PTCA, Daily: 0.745(0.440–0.845); 3x: 0.245 (0.140–0.330); 1x: 0.075(0.020–0.135); 0x: 0.002 (0.000–0.005)].

Selection of a threshold value for PTCA-normalized MVC signal abundance for binary classification of adherence (adherent to regimen vs. not adherent to regimen) was determined from a receiver operating characteristic curve. Fig 1D shows the relationship between MVC/PTCA threshold values and the true positive rate (sensitivity) and true negative rate (specificity) of binary classification. Given the importance of high specificity to avoid damaging patient motivation by mischaracterizing adherence as poor [18], we selected a threshold value of MVC/PTCA = 0.35 (specificity: 100%; sensitivity: 75%) to differentiate 3 or fewer doses per week from more frequent dosing behavior prioritizing high specificity to minimize Type II classification errors that would incorrectly label a patient as having been non-adherent to medication.

### Assessing MVC PrEP adherence patterns in HPTN069/ACTGA5305

**Fig 2:**
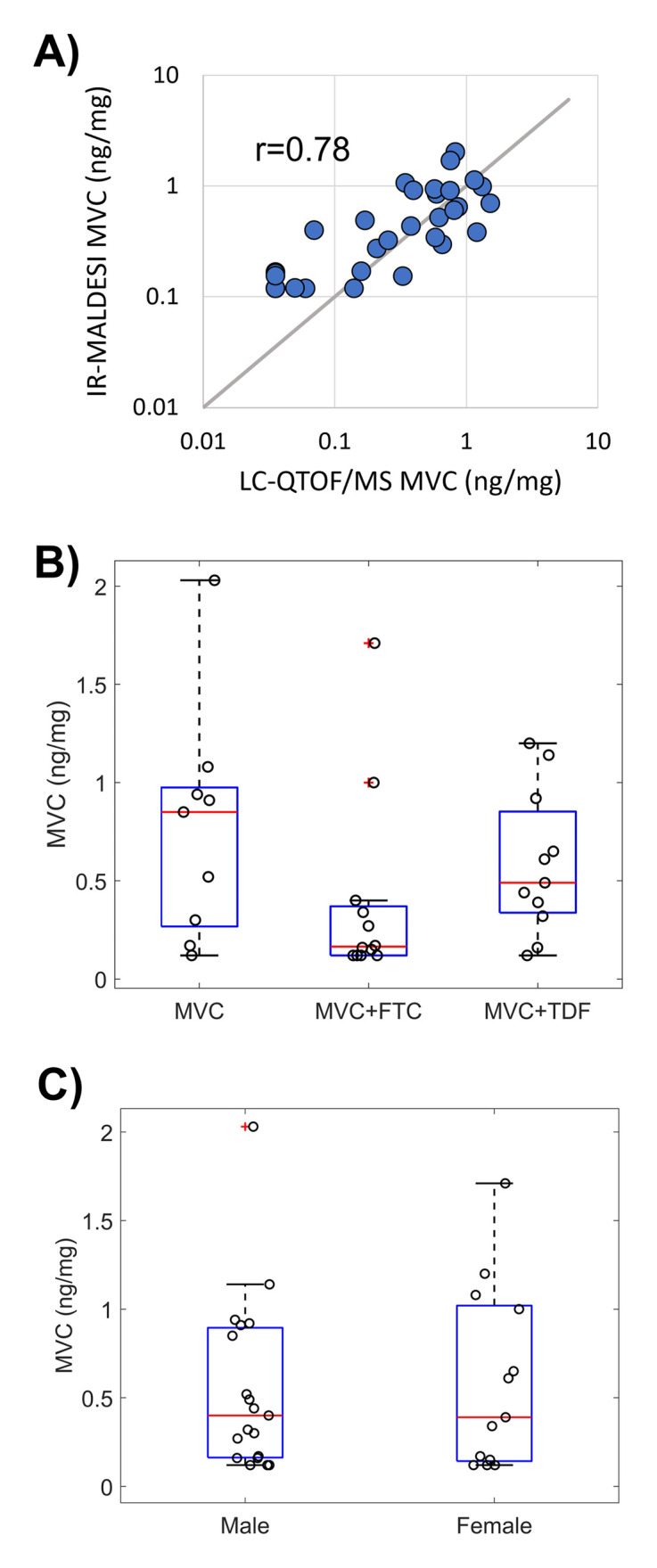
Cumulative MVC accumulation in hair strands during HPTN069/ACTGA5305. (A) Cumulative MVC concentration evaluated in the proximal 1 cm of hair strands by LC-QTOF/MS and MSI. Gray 1:1 line provided for comparison. (B) HPTN069/ACTGA5305 hair substudy MVC concentrations in each dosing arm. (C) HPTN069/ACTGA5305 hair substudy MVC concentrations by sex.

Cumulative MVC concentrations in hair strands collected from across the three HPTN069/ACTGA5305 hair sampling timepoints of a pharmacologic substudy [19] (on drug: Week 24 and 48; follow-up: Week 49; Table 1) were found to be strongly correlated between measurements by MSI and liquid chromatography-quadrupole time-of-flight mass spectrometry (LC-QTOF/MS) (Fig 2A; Spearman’s rho, r = 0.78, P<0.0001). MVC detectability (MSI and LC-QTOF/MS: 26/32 measurable samples), medians (MSI: 0.394 ng/mg, LC-QTOF/MS: 0.361 ng/mg) and ranges (MSI: 0.120–2.03 ng/mg, LC-QTOF/MS: 0.035–1.53 ng/mg) were similar across methods over matched hair segment lengths. We found no difference in MVC concentration among HPTN069/ACTGA5305 regimen arms that included MVC (Fig 2B; P>0.14) or between sexes (Fig 2C; P = 0.94).

**Table 1:** Summary of investigated HPTN069/ACTGA5305 hair samples.

	Study Week			Longitudinal
	*On drug*		*Follow-up*	Samples from
Dosing Arm	24	48	49	Individual Patients
*Maraviroc*				
Male	2	2	2	2
Female	2	1	0	0
*Maraviroc+Emtricitabine*				
Male	4	2	0	2
Female	3	2	1	1
*Maraviroc+Tenofovir Disoproxil Fumarate*				
Male	4	1	2	1
Female	1	1	2	1
Total	16	9	7	7

**Fig 3:**
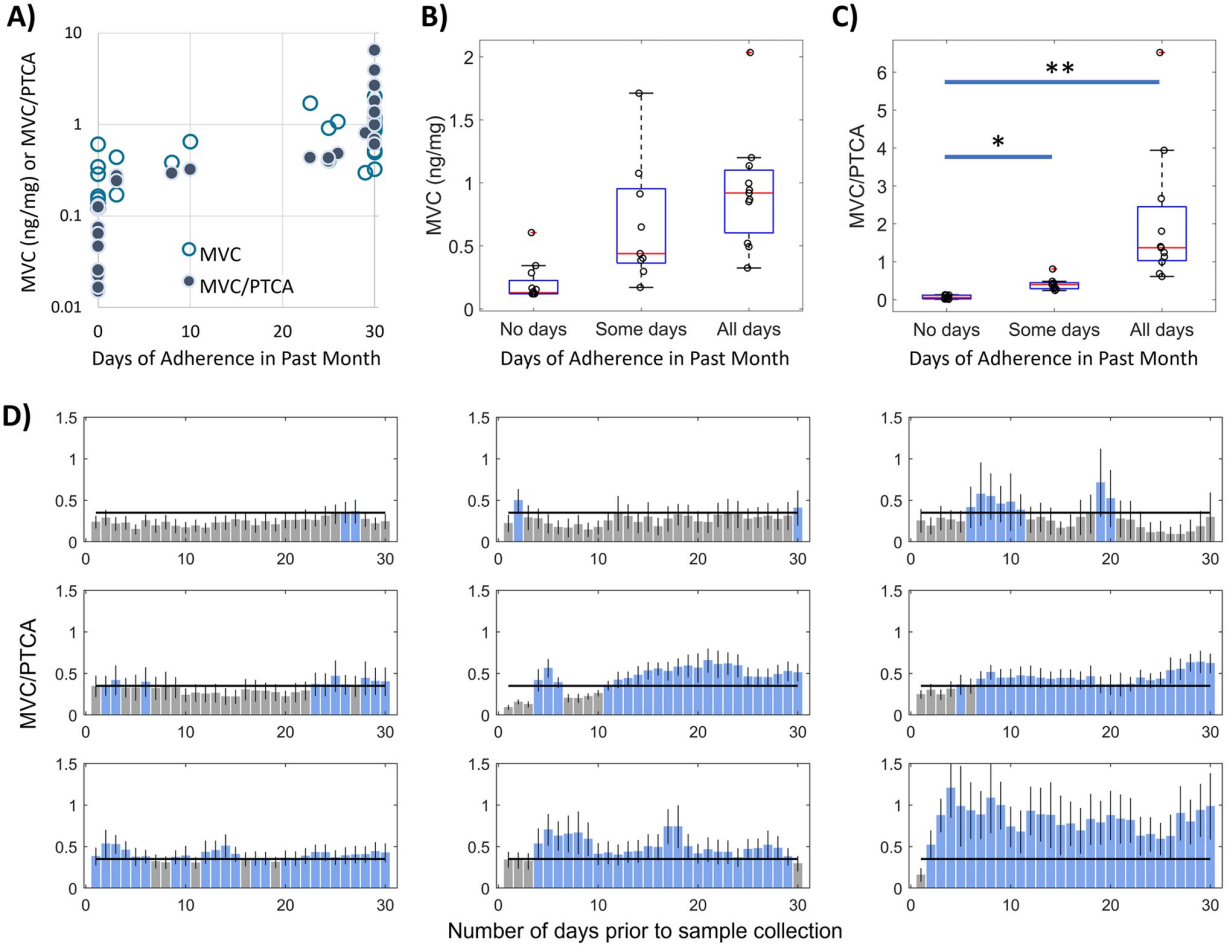
Adherence classification of HPTN069/ACTGA5305 hair strands. (A) MVC concentration or MVC/PTCA normalized response in hair strands relative to the number of days classified as reflecting adherence within the past 30 days prior to hair sample collection. (B) MVC concentration of hair strands within groups of adherence behavior associated with no, some, or all days classified as adherent. (C) MVC/PTCA response in hair strands within groups of adherence behavior associated with no, some, or all days classified as adherent. (D) Bar graphs of adherence measured by MSI within samples where “some days” of adherence was determined. Blue bars reflect days in which MVC response exceeded the adherence cutpoint and gray bars reflect days where MVC response did not exceed the adherence cutpoint.

Using the MVC/PTCA threshold, classification of adherence behavior among the participants (S2 Table) over the prior 30 days fell into three groups: drug response consistent with no days of adherence (n = 12 blue points on left side of Fig 3A; hereafter referred to as “no days”); drug response consistent with some days of adherence (n = 9 blue points across center of Fig 3A; hereafter referred to as “some days”); and, drug response consistent with all days of adherence (n = 11 blue points on right side of Fig 3A; hereafter referred to as “all days”). As with the findings during benchmarking, we further see in Fig 3A that such classification groupings are more ambiguous by MVC concentration alone: a cumulative hair concentration of 0.61 ng/mg, for example, was measured in samples classified separately as having 0/30 and 30/30 days of adherence, respectively. The cumulative MVC concentrations among samples classified as having some days of adherence are not significantly different from those with all days of adherence (Fig 3B; P = 0.61). Conversely, normalization of MVC by PTCA results in MVC/PTCA distributions that are distinguishable across adherence groups (Fig 3C; no days:some days, P = 0.028; no days:all days, P < 0.001; and, some days: all days, P = 0.067). The analysis of daily adherence behavior made possible through IR-MALDESI MSI also reveals variation in the individual patterns of normalized drug responses–including numbers of consecutive days of non-adherence–over the 30-day observation period among the group of samples with “some days” of adherence (Fig 3D). Accompanying bar graphs for samples categorized as “no days” and “all days” of adherence are provided in S2 and S3 Figs, respectively.

**Fig 4:**
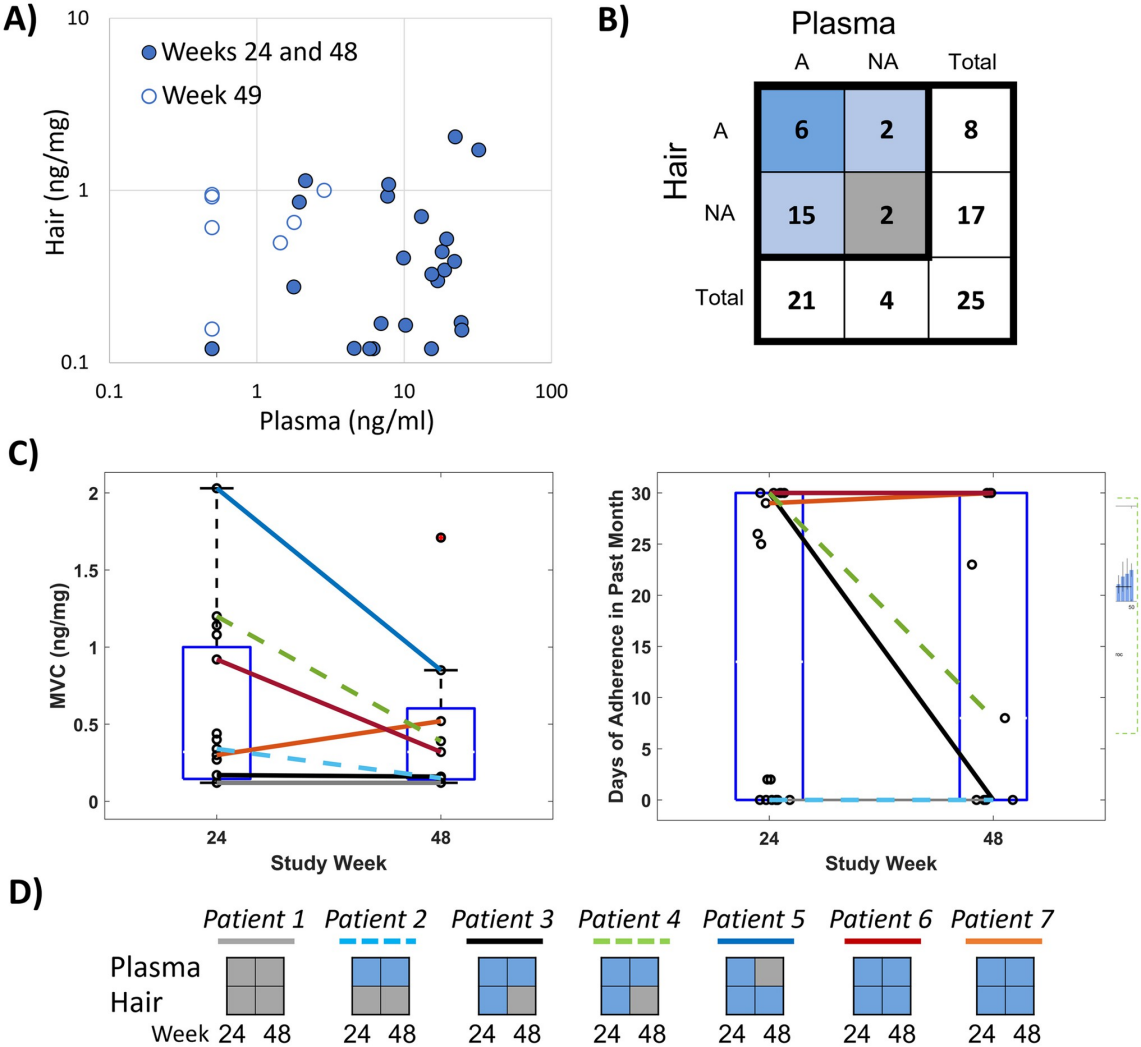
Comparison of long-term and short-term objective adherence measures from HPTN069/ACTGA5305. (A) Comparison of plasma concentration and hair MSI concentration. Samples collected at week 24 and 48 are denoted by a solid circle and samples collected at week 49 have an open circle. (B) Contingency table of adherence classification by hair and plasma where *A* denotes the number of samples classified as adherent and *NA* denotes the number of samples classified as non-adherent. Table shading reflects agreement between measurements where blue corresponds to matched classification of adherence, light blue corresponds to discordance in adherence classification, and gray corresponds to matched classification on non-adherence. (C) Comparison of hair MSI MVC concentration (left) and adherence assessment (right) for on-drug samples collected from individuals at both weeks 24 and 48. Patients with samples at both weeks (n = 7) are denoted by a colored line that is solid (male) or dashed (female). (D) Comparison in longitudinal adherence classification (blue, adherent; gray, non-adherent) between hair and plasma for 7 patients with both types of samples available at each visit.

We found that the cumulative concentrations of MVC in hair, reflecting longer-term behavior over the prior month, had poor correlation with the short-term measure of plasma concentrations of MVC in matched samples when participants were on study drugs (weeks 24 and 48) and at follow-up (week 49) (Fig 4A; r = -0.07, P = 0.72). The correlation between drug concentrations in hair assessed via LC-QTOF/MS to the same plasma MVC measures was similarly low (r = -0.03, P = 0.87). Correlation between these measures improved when comparing IR-MALDESI MSI and plasma in weeks that participants were on drug (i.e., weeks 24 and 48) to account for the rapid MVC clearance from plasma after PrEP discontinuation, which occurred at week 49 (r = 0.40, P = 0.05). Further comparison of hair and plasma results at 24 and 48 weeks reveals disagreement in binary classification of adherence (Fig 4B), with 84% of samples (21/25) classified as adherent by the plasma MVC threshold of 4.6 ng/ml [19] and only 32% of samples (8/25) classified as adherent according to IR-MALDESI. Agreement between the two classification measures occurred in only 32% of samples, and when the classification for a sample disagreed it was most often the case that the sample was classified as adherent based on plasma and non-adherent based on hair analysis (88% of discordance; McNemar test: χ^2^ = 9.94, P = 0.0016). Comparison of whole-cohort MVC concentrations in hair at week 24 vs. week 48 (Fig 4C, left) shows no statistically significant difference between time points (P = 0.73). In the seven participants for whom data were available at both 24 and 48 weeks, one patient had an apparent increase in MVC concentration between week 24 and 48, two had low concentrations at both time points, and four had a decrease in hair concentrations. As with Fig 3C, we find that normalization to account for different melanin levels reveals different patterns from those in unadjusted analyses (cf. right panel vs. left panel of Fig 4C): here, three participants appear to have similar levels of adherence at 24 and 48 weeks, two have consistent non-adherence, and two have decreasing adherence. For these seven participants, agreement between plasma and MSI hair adherence classification at 24 and 48 weeks varied from total discordance to total concordance (Fig 4D).

Poor agreement was also found between frequency of pill openings within the prior month measured by Wisepill electronic monitoring and pharmacologic measures of adherence in 15 samples with matched records (S4 Fig). Spearman’s rho relating pill openings to hair MVC concentrations (r = 0.12, P = 0.68) and plasma (r = -0.07, P = 0.81) suggest low correlation between paired values.

## Discussion

In this work, we have demonstrated the capability of IR-MALDESI MSI to evaluate MVC longitudinally in single hair strands at high spatial resolution and classify short-term changes in adherence behavior over the longer-term drug dosing record provided by hair. Accounting for differences in the accumulation of MVC in hair based on its melanin content was necessary in order to unambiguously classify adherence in the ENLIGHTEN study. Correlation between melanin and concentration of drug in hair has been well documented in forensic toxicology analysis of illicit drugs [20, 21], where the effect is stronger for basic compounds. Our prior work showed that a biomarker of melanin, PTCA, was more strongly correlated with MVC accumulation in hair than more acidic antiretrovirals such as emtricitabine and dolutegravir and suggested that the binding of MVC to melanin may limit its removal from hair strands after chemical hair treatments [17]. Binary classification of adherence based on MVC/PTCA normalization was selected to maintain assay applicability across varied hair colors and types, and we prioritized assay selectivity in establishing a MVC/PTCA threshold cutpoint differentiating 3 or fewer doses/week from more frequent dosing. This was based on feedback from patients and providers indicating it was crucially important to avoid a false designation of non-adherence and preserve patient trust and confidence [18]. As a result, adherence classification based on the selected cutpoint (MVC/PTCA = 0.35) can be considered conservative for differentiating daily and intermittent dosing. Since target concentrations of MVC for PrEP have not been defined, it is important to note that this threshold cutpoint only reflects adherence rather than efficacy.

Longer-term adherence in HPTN069/ACTGA5305 from hair strands provided a considerably different perspective of adherence than the interpretation of matched plasma samples collected at the same timepoints [19]. Our classification of daily adherence by IR-MALDESI MSI indicated that less than one-third of samples reflected consistent, sustained adherence throughout the prior 30 days. The corresponding plasma MVC concentrations, with an elimination half-life of 16 hours, indicated much higher adherence (84%) based on classification using the adherence threshold defined conservatively in the pharmacologic substudy relative to benchmarked TFV and FTC plasma adherence thresholds (specificity: 78%; sensitivity: 93%). This higher adherence was consistent with the characterization of adherence from plasma in the whole cohort in both cis-gender women (79%) and men who have sex with men (90%) [19]. Although these samples were collected at the same visit, they do not necessarily represent the same period of drug exposure. This is because the hair samples were not plucked to include the follicle, but rather cut close to the scalp, meaning the most recent time point reflected by the proximal end of a hair sample may correspond to growth occurring more than a week before collection [22]. As such, these comparisons have been interpreted solely as differences in short-term and longer-term adherence behavior. The discordance in adherence classification observed, with matched samples predominately classified as adherent by plasma and having less than full adherence by hair strands, likely arises from changes in adherence behavior just prior to a clinic visit such as white-coat adherence [23] or participants simply being sensitized to the study and related procedures around study visits. The discordance between these measures underscores the need for monitoring both short and longer-term adherence as part of an approach to evaluate PrEP efficacy.

Findings of longer-term adherence captured in hair via IR-MALDESI MSI were consistent with an additional pharmacologic assessment in the HPTN069/ACTGA5305 tissue sub-study. A comparison of the interquartile range of rectal concentrations of PrEP antiretrovirals (MVC, TFV, and FTC) [19] with pharmacokinetic studies of rectal drug concentrations ranging from single dose to steady-state dosing [24–26] (S3 Table) indicates that median tissue concentrations from individuals sampled across each dosing arm of HPTN069/ACTGA5305 fell below median values expected from daily dosing and may be more consistent with 4 or fewer doses per week. The average level of adherence suggested by these tissue concentrations corresponds with our IR-MALDESI findings but not the high adherence indicated by the plasma results.

Hair strand MSI analyses indicated heterogeneous periods of active dosing over 30 days where at least periodic engagement with PrEP was quantifiable in all but 5 samples. This approach offers a unique ability to evaluate both short-term and longer-term changes that are not captured by Wisepill, the data from which were not well-correlated with any other pharmacologic measures of adherence in HPTN069/ACTGA5305. It is important to note that none of the hair samples investigated here came from any of the 6 individuals who seroconverted during HPTN069/ACTGA5305 [15]. While the number of individuals for whom we were able to evaluate samples collected from both timepoints during active PrEP dosing in the HPTN069/ACTGA5305 study was limited, we found significant differences in adherence patterns that remained either high or low, or decreased over the course of the study. Although reasons behind non-adherence are varied, some patterns of use identified by hair MSI may have been a prevention-effective strategy [9], whereby study subjects took PrEP during periods of potential exposure to HIV, or perceived risk of HIV. A recent investigation of sexual behavior among MSM within the HPTN069/ACTGA5305 cohort indicated that participants reporting condom-less sex had higher rates of plasma drug concentrations classified as adherent [27]. Assessing adherence in the context of risk behavior may provide an important mechanism to support and sustain adherence and persistence of PrEP use.

Both patients and providers have previously found our visual display of IR-MALDESI MSI adherence patterns to be informative [28]. This time-series of adherence offers a measurement that can be correlated with dynamic assessments of risk to determine the potential effectiveness of individual adherence patterns. Even as long-acting methods of PrEP delivery become more common as an alternative to daily oral dosing, the longer-term drug concentration information provided by MSI of hair may be useful in monitoring the tail of long-acting drug availability to ensure protection.

Our study has several limitations. The sample size of our benchmarking study was small, which allowed us to identify a threshold differentiating daily dosing from 3 or fewer doses per week but further studies will be needed to discriminate dosing frequency more granularly. Collection of hair strands by cutting precluded interrogation of an individual’s most recent drug-taking behavior, and we recommend plucking 5 strands when an assessment of the most recent week of dosing is essential. Finally, the availability of HPTN069/ACTGA5305 hair samples also limited the number of individuals who participated in the pharmacokinetic sub-study that we could investigate.

We have shown the unique capabilities of IR-MALDESI MSI for evaluating daily antiretroviral adherence throughout the record of drug accumulation preserved in hair strands. While MVC has not moved forward as a PrEP candidate, our work underscores the importance of including a long-term measure of adherence as part of an adherence assessment in trials of future PrEP candidates. The multi-faceted pharmacology data collected during HPTN069/ACTGA5305 uniquely allowed us to correlate long-term assessment of adherence in hair to tissue data, further supporting the long-term assessment of adherence in hair samples that can be collected non-invasively. Additionally, our work suggested the importance of evaluating hair melanin content when making comparisons in the accumulation of basic and lipophilic compounds like MVC between patients. IR-MALDESI MSI offers a new approach for measuring adherence patterns that provides a temporal overview of dosing that can be used in research and could be an important addition to adherence monitoring and intervention. The approach is sensitive to a range of ARVs and other small molecules [29] making it highly adaptable for monitoring multidrug regimens, including those containing FTC as we have demonstrated previously [30].

## Materials and methods

### Study design

Benchmarking of MVC in hair strands was performed as part of the ENLIGHTEN Study (NCT03218592), conducted at the North Carolina Translational and Clinical Sciences (NC TraCS) Institute Clinical and Translational Research Center (CTRC). HIV-uninfected healthy volunteers (n = 12, S1 Table) aged 18 to 70 years of age, inclusive on the date of screening, with an intact gastrointestinal system and at least 1cm caput hair were administered MVC 300mg by directly observed therapy. The selected sample size was calculated to be sufficient to achieve >99% power to establish dose proportionality. All study volunteers participated in a 28-day period of daily dosing after which they were randomized (n = 4) for a subsequent 28-day period to one of three differentiated dosing frequencies: 0 doses/week, 1 dose/week, or 3 doses/week. An interval of 7 days separated each dosing period. This enabled characterization of MVC accumulation in hair strands by IR-MALDESI MSI under different dosing patterns. Hair sampling was conducted by cutting approximately 10 hair strands from the occipital region close to the scalp using scissors. Strands were adhered to aluminum foil at their distal end to preserve orientation before being sealed and stored with a desiccant gel pack at 4°C until analysis. Hair was collected during study visits on Day 3, 7, 14, 21 and 28 of each dosing period. MSI response to MVC accumulation in ENLIGHTEN hair strands in daily and intermittent dosing periods was conducted using samples collected at the end of daily and intermittent dosing phases.

Characterization of PrEP adherence by IR-MALDESI MSI was performed through HPTN069/ACTGA5305 (NCT01505114). This was a 48-week placebo-controlled study in at-risk MSM and women of the safety and tolerability of candidate HIV PrEP regimens including MVC alone or in combination with either tenofovir disoproxil fumarate (TDF) or emtricitabine (FTC) in comparison to TDF+FTC [15, 16]. To complement the objective adherence measurements undertaken for all participants (electronic drug monitoring using a single pillbox (Wisepill) containing the 3 ARVs, drug level monitoring from blood stored at every visit), additional sampling of tissues, plasma, and hair was conducted through a nested pharmacologic substudy described elsewhere [19]. Approximately 200 strands of hair were collected from sub-study participants at three time points (on drug: Week 24, Week 48; follow-up: Week 49). Hair storage followed the same protocol as the ENLIGHTEN study. IR-MALDSI MSI analysis was performed on all samples from MVC-based regimens (MVC, MVC+TDF, MVC+FTC) for which hair was not consumed during LC-QTOF/MS analysis. As summarized in Table 1, this corresponded to a total of 32 samples collected across the study from 19 participants (10 male, 9 female) whose demographic information is included in S2 Table.

### Ethics statement

The ENLIGHTEN study was granted ethical approval by the University of North Carolina at Chapel Hill Institutional Review Board. All participants provided written informed consent before enrollment. HPTN069/ACTG5305 samples were provided under approved request from the HPTN069/ACTG5305 study team and additional approval or consent was not required.

### Hair analysis

#### IR-MALDESI MSI

Hair strands (n = 4) were oriented horizontally and adhered to glass microscope slides with proximal strand ends positioned to the left for analysis by IR-MALDESI MSI [29, 31]. Prepared sample slides were positioned on a temperature-controlled stage in the IR-MALDESI MSI source enclosure before being cooled to -9°C under dry nitrogen gas flow to reduce humidity. Following temperature stabilization, the nitrogen flow was interrupted and the MSI source was opened to the ambient atmosphere to grow a thin layer of ice on the sample surface. Following ice growth, the source was closed and nitrogen was used to maintain a relative humidity of ~14% throughout the experiment to preserve ice thickness. The ice layer promoted sample desorption from single IR laser pulses (λ = 2940 nm, IR Opolette, Opotek, Carlsbad, CA). Volatilized material expanding upward from the sample intersected an orthogonal electrospray plume to create analyte ions, which were sampled into an orbitrap mass spectrometer (ThermoFisher Q Exactive Plus, Bremen, Germany) for analysis. A list of targeted analytes is shown in S4 Table. For analysis of positive ions, the mass spectrometer was operated in positive polarity full scan mode (m/z 200 to 800; resolving power: 140,000 at m/z 200; s-lens RF level: 50, mass accuracy: <1 ppm). For analysis of negative ions, the mass spectrometer was operated in full scan mode with negative polarity (*m/z* 190–760; resolving power: 140,000 at *m/z* 200; s-lens RF level: 50, mass accuracy: <1 ppm). Analysis was performed with a step-size of 100 μm between sampling locations, corresponding to approximately 7–8 hours of growth based on the average growth rate (~ 1cm/month) in the occipital region [22]. The MVC response from three neighboring sampling locations along a composite longitudinal profile of sampled hair strands was binned to evaluate daily accumulation of drug in hair throughout the entire period of assessment (benchmarking: 1.5 cm, 1.5 months; HPTN069/ACTGA5305: 1 cm, 1 month).

Calibration of IR-MALDESI response to MVC in hair strands was performed using standards prepared from blank (drug-free) hair matrix by incubation in drug. Standards were prepared by transferring drug-free hair (approximately 10 mg) into a vial containing 20 mL of analyte and solvent (50:50 Methanol:Water), and incubating for approximately 24 hours in a reciprocal shaking bath. Incubated standards were then rinsed with fresh solvent and stored at -20°C and used as needed for analysis. MVC concentrations of incubated standards were determined by LC-MS/MS, covering the range 0.145–2.99 ng/mg hair. One level of standards (0.299 ng/mg) was reserved for use as a positive control in all assessments of clinical samples. A representative image showing the MVC response from a calibration and a composite calibration curve from n = 5 calibrations conducted during experimental work is shown in S5 Fig.

Separate regions of interest were interrogated by IR-MALDESI MSI for analysis of MVC and PTCA in clinical samples (S6 Fig). MVC was evaluated in the proximal 1 cm of samples, corresponding to the most recent growth of hair prior to sampling, and cumulative concentrations of MVC were determined by averaging MSI signal abundance over this segment length for comparison to LC-QTOF/MS. This region of interest also included two vertically-oriented positive control strands. Because rastering during MSI analysis proceeds from left-right, top-to-bottom, positive control strands oriented vertically provided an assessment of MVC sensitivity throughout data acquisition. The melanin biomarker, PTCA, was evaluated distally in sample hair strands by submerging this end of the slide in a solution of 1 M ammonium hydroxide in 45/45/10 methanol/water/hydrogen peroxide (v/v/v) for 10 min. Normalized MVC/PTCA profiles were then compared to the adherence threshold for daily adherence classification.

Data were processed using freely available MSiReader and custom MATLAB software (Mathworks, Inc., Natick, MA). Raw mass spectrometry files were converted into the imZML file structure that could be interrogated by MSiReader to extract the ion heatmaps delineating ions of interest along the hair strands with a mass window of 5 ppm. A custom MATLAB interface evaluated longitudinal profiles of MVC response in individual strands within a sample, which were then aligned to a common reference and averaged radially to create a composite sample profile.

#### LC-QTOF/MS

LC-QTOF/MS analysis of MVC in HPTN069/ACTGA5305 hair samples was conducted in the UCSF TB Hair Analysis Laboratory. Hair strands (proximal 1 cm segments, 2 mg) were pulverized using an Omni Bead Ruptor homogenizer (OMNI International, NW Kennesaw, GA, USA). Pulverized hair was extracted with 0.5 mL methanol followed by a two-hour mixing in a water bath shaker maintained at 37°C; the resulting extract was evaporated before reconstitution to 0.2 mL 10% acetonitrile in water with 0.1% formic acid. The sample extract (5 μL) was injected into the Agilent Liquid Chromatograph 1260 (Agilent Technologies, Sta Clara, CA) attached to an Agilent Quadrupole Time-of-Flight Mass Spectrometer 6550. Analytes in the sample extract were separated by gradient elution on an Agilent Poroshell 120, EC-C18 column (2.1 x 100 mm, 2.7 μm particle size) using water with 0.05% formic acid and 5mM ammonium formate as mobile phase A (MPA) and acetonitrile with 0.05% formic acid as mobile phase B (MPB). The gradient used for analyte separation consisted of 5% MPB at 0–0.5 min, gradient to 30% MPB from 0.5 to 1.5 min, gradient to 70% MPB from 1.5 to 4.5 min, gradient to 100% MPB at 4.5–7.5 min, and 100% MPB at 7.5–10 min; a post-wash at 5% MPB followed each run for 4 min. Ionization of MVC in the mass spectrometer was achieved using electrospray ionization (ESI) in positive polarity, and data acquisition was performed in the auto- MS/MS mode. Detection of the analyte was done by accurate mass match within 10 parts per million, retention time match within 0.1 min, target score (indicator of isotopic pattern match) of at least 70 and an MS/MS spectral match score of at least 70.

Quantification of MVC was done by isotope dilution method using MVC-d6 as internal standard. MVC drug levels were normalized by weight. The limit of detection was 0.05 ng/mg while the lower limit of quantification was 0.2 ng/mg. Procedural quality control materials and procedural blank were run along with the calibration curve at the start, middle, and end of each run. Two quality control materials were used at low and high concentrations. To accept the results of a batch run, QC materials measurements must be within 15% of their target values.

### Statistical analyses

Nonparametric statistical tests were selected based on sample size. The Wilcoxon rank-sum test was used to compare two experiment groups. Spearman’s rank order correlation (Spearman’s rho, rs) was used to assess the relationship between two analytical measures of matched samples. A Kruskal-Wallis one-way analysis of variance (ANOVA) test followed by Dunn-Sidak p-value corrections for multiple comparisons was performed between three experiment groups. Statistical significance by these methods was obtained by using Matlab. McNemar’s test of paired nominal data was used to compare binary adherence classifiers, obtained from R.

## Supporting information

S1 Fig Profiling MVC concentrations longitudinally in hair strands. A-F) Time series of MVC measured by IR-MALDESI MSI in hair strands from an individual in the ENLIGHTEN study collected at the end of daily dosing and throughout intermittent (1x/week) dosing. G) Corresponding average longitudinal profiles for each IR-MALDESI image in A-F showing the distal shift of the daily dosing MVC signal abundance with continued hair growth.
(TIF)

S2 Fig Bar graphs of daily adherence classification measured by MSI within samples where “no days” of adherence was determined. (TIF)

S3 Fig Bar graphs of daily adherence classification measured by MSI within samples where “all days” of adherence was determined. (TIF)

S4 Fig Comparison between Wisepill frequency of pill openings detected within the past 30 days and MVC concentrations in hair and plasma. A) Correlation with hair MVC concentrations measured by IR-MALDESI MSI; B) Correlation with proportion of the past 30 days classified as adherent by MSI; C) Correlation with hair MVC concentrations measured by LC-QTOF/MS; and, D) Correlation with plasma MVC concentrations. Spearman correlation coefficient and probability for each measure are reported in inset.
(TIF)

S5 Fig Calibration of IR-MALDESI MSI response to MVC in hair. A) Relationship between MVC concentration of incubation supernatant and MVC concentration measured in the resulting hair reference standards by LC-MS/MS. B) Example IR-MALDESI MSI ion map of MVC from calibration of using incubated hair strands. Vertically oriented strands correspond to positive quality control standards. C) Average calibration response (blue dots) from n = 5 calibrations conducted over the course of HPTN069/ACTGA5305 sample analysis. Also shown is the average response of the positive quality control standards (orange) measured during analysis of each clinical sample (n = 32). Error bars reflect one standard deviation of the measurements.
(TIF)

S6 Fig Schematic of IR-MALDESI MSI analysis of HPTN069/ACTGA5305 sample strands. Positive quality control strands (n = 2) were oriented vertically next to the proximal end of sample strands (n = 4). MVC was measured with a region of interest including positive controls and the proximal end of hair strands (red ROI). PTCA was measured in a separate distal region of interest of the same sample strands (blue ROI).
(TIF)

S1 Table Demographic information for ENLIGHTEN maraviroc DOT study participants. (DOCX)

S2 Table Demographic information for HPTN069/ACTGA5305 participants providing hair samples for evaluation by IR-MALDESI MSI and LC-QTOF/MS. (DOCX)

S3 Table Comparison of colorectal ARV tissue concentrations in HPTN069/ACTGA5305 and pharmacokinetic studies. (DOCX)

S4 Table IR-MALDESI MSI analytes targeted in hair strand analysis. (DOCX)
