# Supplementary figures and images for: Mass spectrometry imaging of hair identifies daily maraviroc adherence in HPTN 069/ACTG A5305
Source: PLoS One. 2023 Jun 23;18(6):e0287449. doi: 10.1371/journal.pone.0287449 (PMC10289441; doi:10.1371/journal.pone.0287449)

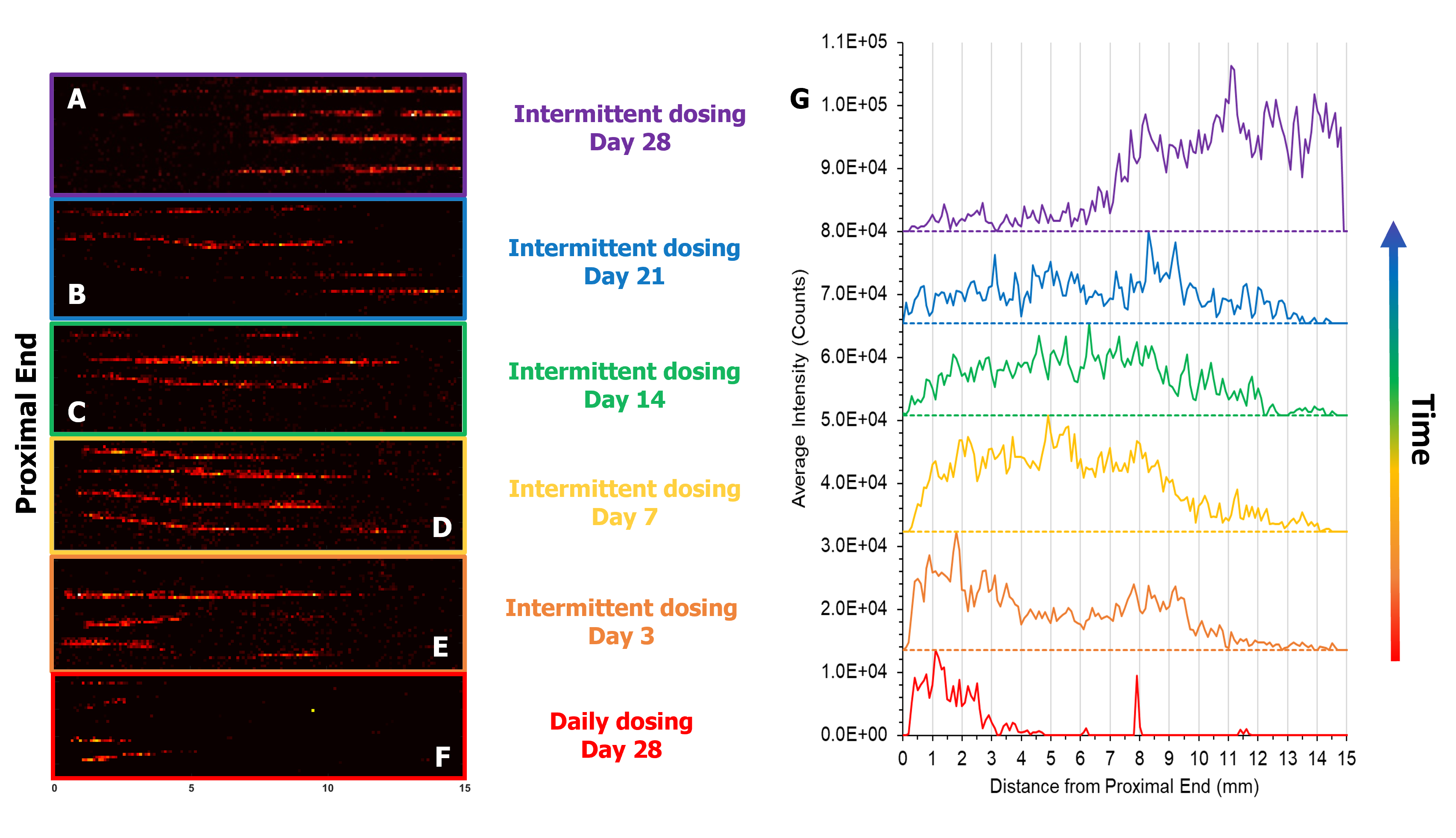

Supplement: S1 Fig — A-F) Time series of MVC measured by IR-MALDESI MSI in hair strands from an individual in the ENLIGHTEN study collected at the end of daily dosing and throughout intermittent (1x/week) dosing. G) Corresponding average longitudinal profiles for each IR-MALDESI image in A-F showing the distal shift of the daily dosing MVC signal abundance with continued hair growth. (TIF) [file pone.0287449.s001.tif]

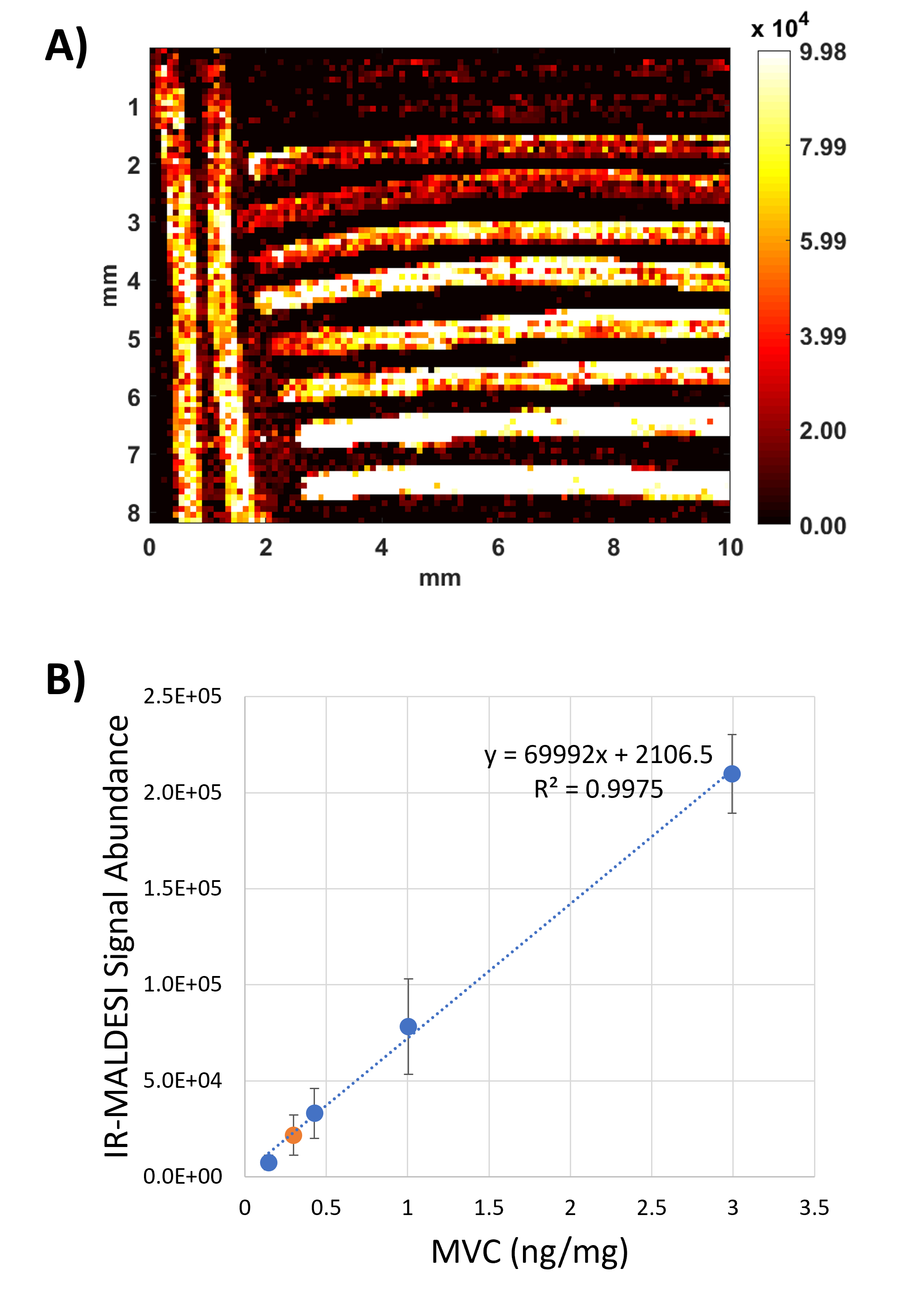

Supplement: S2 Fig — (TIF) [file pone.0287449.s002.tif]

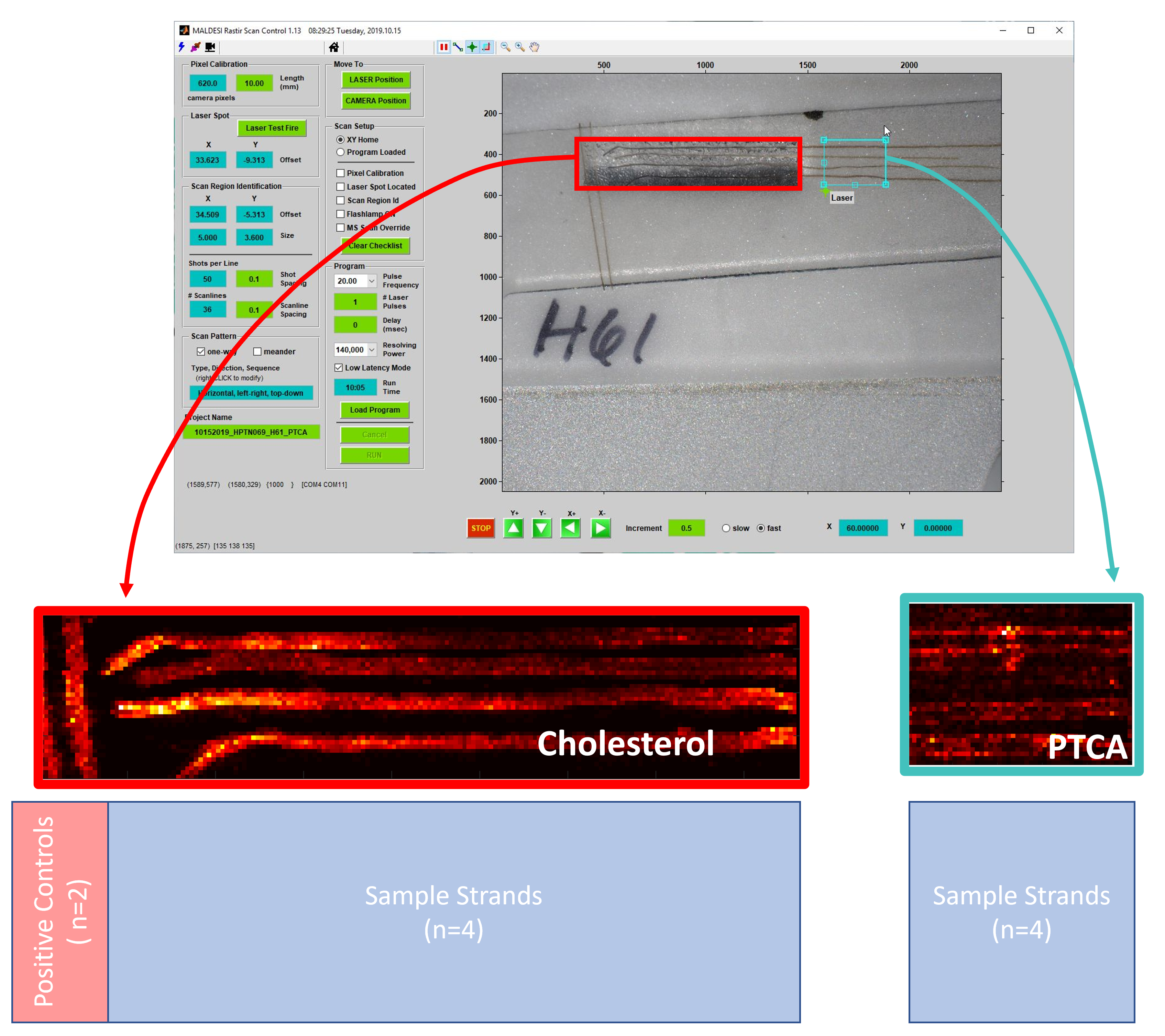

Supplement: S3 Fig — (TIF) [file pone.0287449.s003.tif]

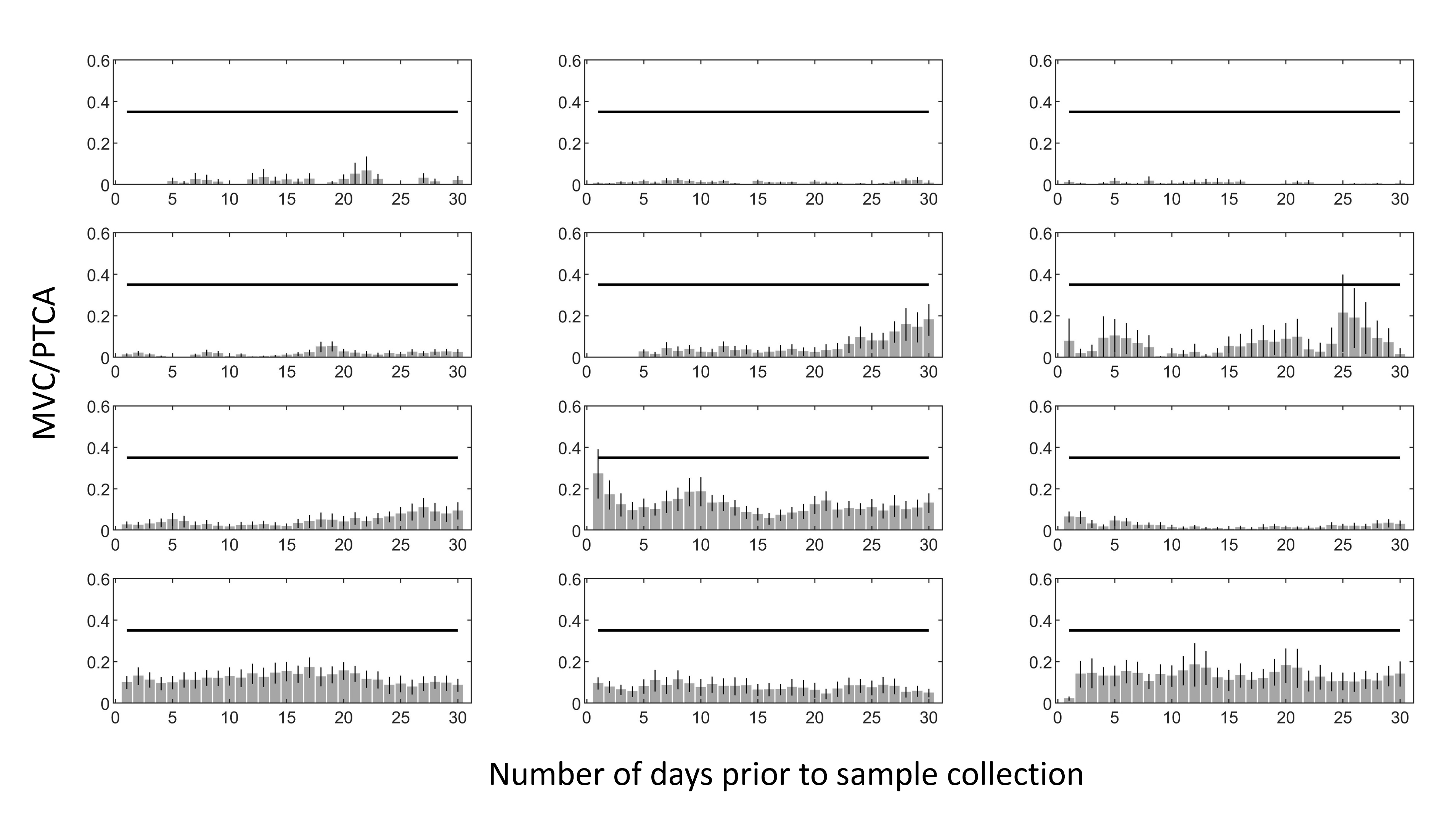

Supplement: S4 Fig — A) Correlation with hair MVC concentrations measured by IR-MALDESI MSI; B) Correlation with proportion of the past 30 days classified as adherent by MSI; C) Correlation with hair MVC concentrations measured by LC-QTOF/MS; and, D) Correlation with plasma MVC concentrations. Spearman correlation coefficient and probability for each measure are reported in inset. (TIF) [file pone.0287449.s004.tif]

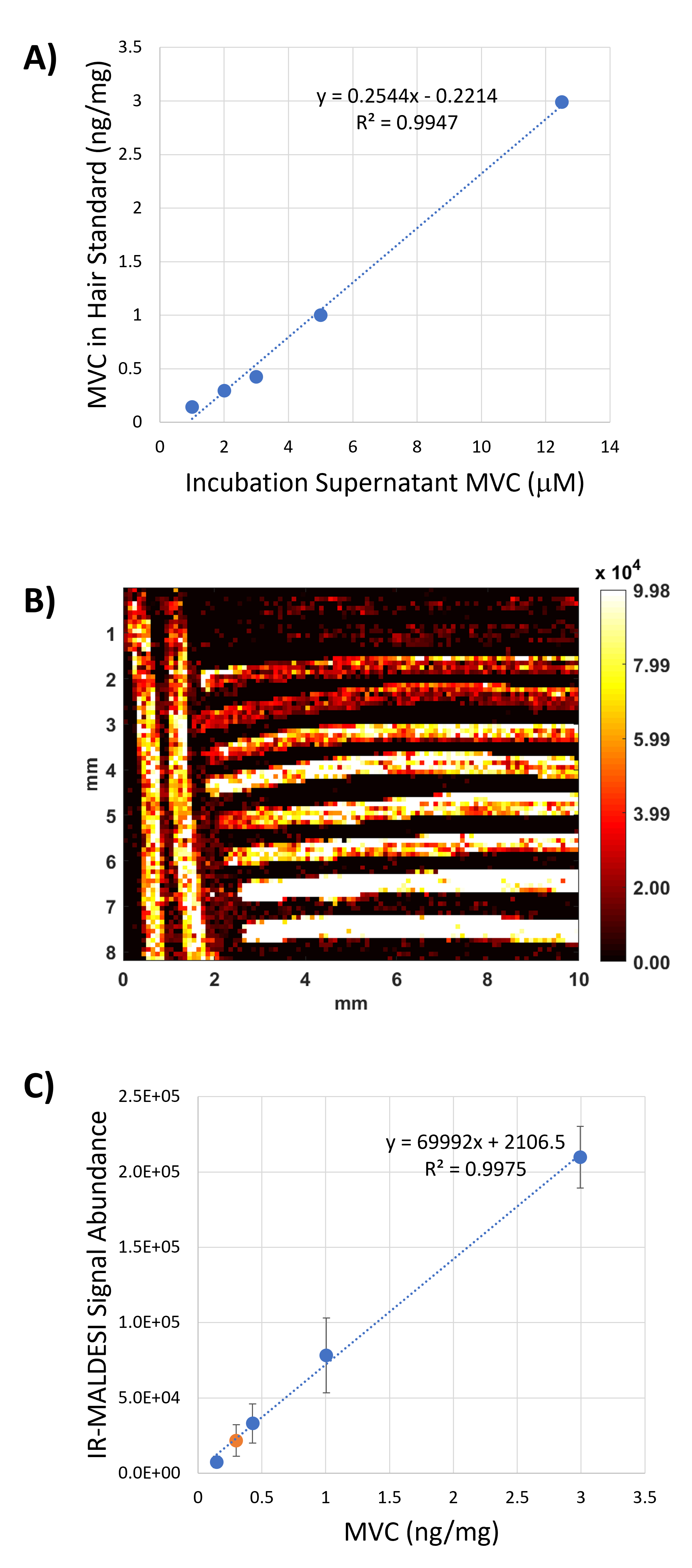

Supplement: S5 Fig — A) Relationship between MVC concentration of incubation supernatant and MVC concentration measured in the resulting hair reference standards by LC-MS/MS. B) Example IR-MALDESI MSI ion map of MVC from calibration of using incubated hair strands. Vertically oriented strands correspond to positive quality control standards. C) Average calibration response (blue dots) from n = 5 calibrations conducted over the course of HPTN069/ACTGA5305 sample analysis. Also shown is the average response of the positive quality control standards (orange) measured during analysis of each clinical sample (n = 32). Error bars reflect one standard deviation of the measurements. (TIF) [file pone.0287449.s005.tif]

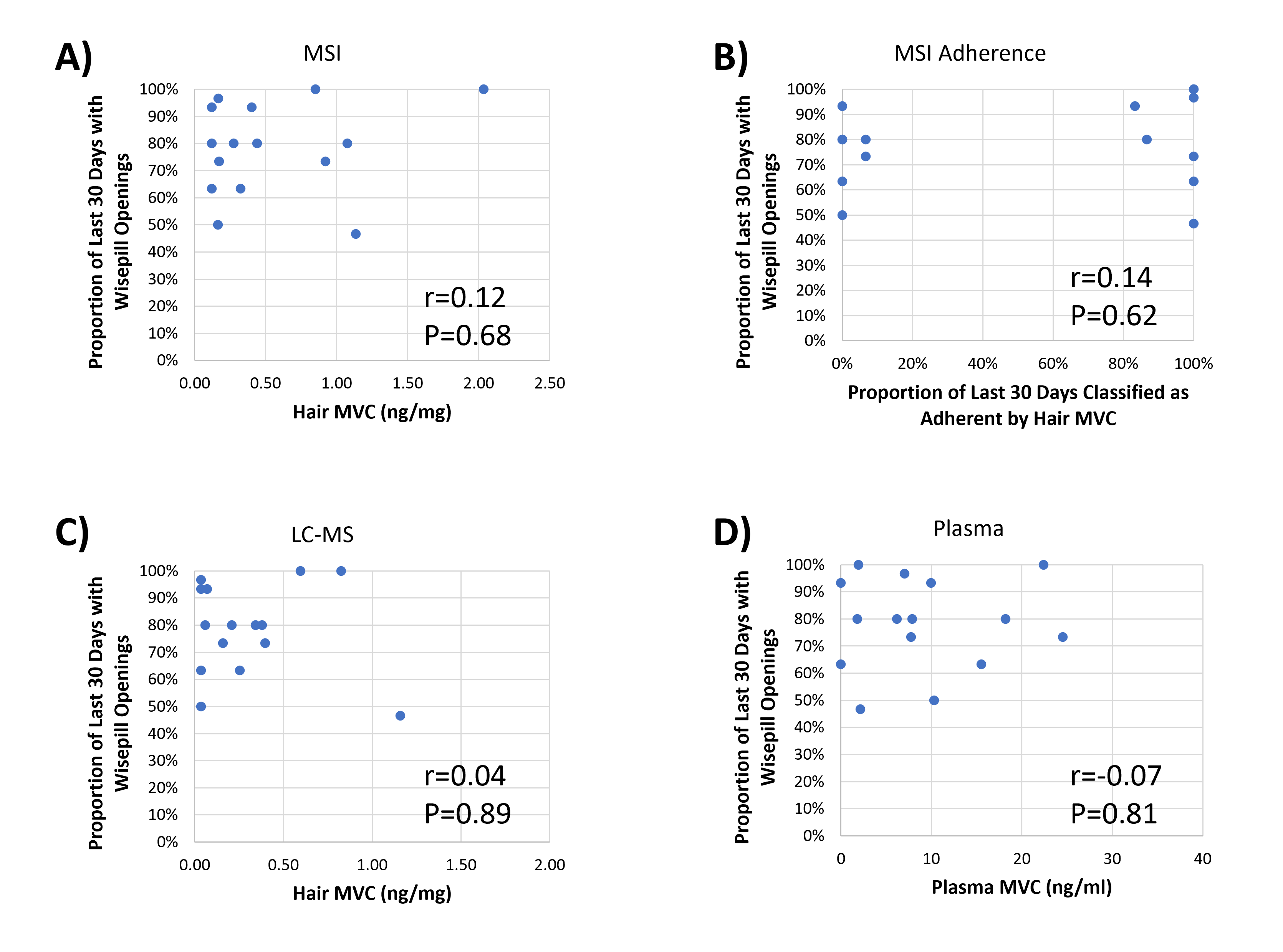

Supplement: S6 Fig — Positive quality control strands (n = 2) were oriented vertically next to the proximal end of sample strands (n = 4). MVC was measured with a region of interest including positive controls and the proximal end of hair strands (red ROI). PTCA was measured in a separate distal region of interest of the same sample strands (blue ROI). (TIF) [file pone.0287449.s006.tif]
