# Supplementary material for: Mass spectrometry imaging of hair identifies daily maraviroc adherence in HPTN 069/ACTG A5305
Source: PLoS One. 2023 Jun 23;18(6):e0287449. doi: 10.1371/journal.pone.0287449 (PMC10289441; doi:10.1371/journal.pone.0287449)
Supplement: S1 Table — (DOCX) [file pone.0287449.s007.docx]

| **Sex** | **Race** | **Ethnicity** | **Age** | **BMI** | **Hair Color** |
| --- | --- | --- | --- | --- | --- |
| Female | Caucasian | Hispanic | 31 | 38.26 | Brown |
| Female | Caucasian | Hispanic | 19 | 20.95 | Blonde |
| Female | Asian | Non-Hispanic | 18 | 24.02 | Brown |
| Female | Caucasian | Non-Hispanic | 23 | 21.34 | Blonde |
| Female | Caucasian | Non-Hispanic | 31 | 31.47 | Pastel Blue |
| Male | Caucasian | Hispanic | 47 | 25.82 | Black |
| Female | Caucasian | Non-Hispanic | 33 | 24.16 | Blonde |
| Female | Caucasian | Non-Hispanic | 29 | 21.57 | Brown |
| Female | Caucasian | Non-Hispanic | 45 | 23.4 | Blonde |
| Female | African American | Non-Hispanic | 45 | 23.12 | Black |
| Female | Caucasian | Non-Hispanic | 20 | 28.27 | Brown |
| Male | Caucasian | Non-Hispanic | 51 | 40.16 | Blonde |

**S1 Table**. **Demographic information for ENLIGHTEN maraviroc DOT study participants.**
