# Supplementary material for: Mass spectrometry imaging of hair identifies daily maraviroc adherence in HPTN 069/ACTG A5305
Source: PLoS One. 2023 Jun 23;18(6):e0287449. doi: 10.1371/journal.pone.0287449 (PMC10289441; doi:10.1371/journal.pone.0287449)
Supplement: S2 Table — (DOCX) [file pone.0287449.s008.docx]

| **Sex** | **Race** | **Ethnicity** | **Age** |
| --- | --- | --- | --- |
| Male | Caucasian | Non-Hispanic | 24 |
| Male | Caucasian | Non-Hispanic | 20 |
| Male | Caucasian | Non-Hispanic | 65 |
| Female | African American | Non-Hispanic | 52 |
| Female | Caucasian | Non-Hispanic | 33 |
| Female | African American | Non-Hispanic | 34 |
| Male | Caucasian | Hispanic | 29 |
| Male | Caucasian | Non-Hispanic | 30 |
| Male | Caucasian | Non-Hispanic | 35 |
| Male | Caucasian | Non-Hispanic | 28 |
| Female | African American | Non-Hispanic | 27 |
| Male | Caucasian | Non-Hispanic | 29 |
| Female | Caucasian | Non-Hispanic | 52 |
| Female | Caucasian | Non-Hispanic | 25 |
| Male | Caucasian | Non-Hispanic | 49 |
| Male | Caucasian | Non-Hispanic | 49 |
| Female | Caucasian | Non-Hispanic | 22 |
| Female | Caucasian | Non-Hispanic | 28 |
| Female | African American | Non-Hispanic | 31 |

**S2 Table. Demographic information for HPTN069/ACTGA5305 participants providing hair samples for evaluation by IR-MALDESI MSI and LC-QTOF/MS.**
