# Supplementary material for: Mass spectrometry imaging of hair identifies daily maraviroc adherence in HPTN 069/ACTG A5305
Source: PLoS One. 2023 Jun 23;18(6):e0287449. doi: 10.1371/journal.pone.0287449 (PMC10289441; doi:10.1371/journal.pone.0287449)
Supplement: S3 Table — (DOCX) [file pone.0287449.s009.docx]

|  |  | **FTC** | **TFV** | **MVC** |
| --- | --- | --- | --- | --- |
| **HPTN069/ACTGA5305** | | 0.7 (0.3-1.1)* | 1.4 (0.8-2.1)* | 0.7 (0.2-1.4)* |
|  |  |  |  |  |
| **Directly observed dosing** | |  |  |  |
|  | 7x/week | 20.5 (1.87-49.32)** | 5.42 (0.21-20.54)** |  |
|  | 4x/week | 1.22 (0.36-5.74)** | 1.58 (0.21-4.75)** |  |
|  | 2x/week | 0.49 (0.2-2.08)** | 0.23 (0.05-3.28)** |  |
|  | 1x/week | 0.13 (BLQ-0.21)** | 0.02 (0.01-0.10)** |  |
|  | Single dose |  |  | 1.19**** |
|  | Steady-state | 11.1 (1.6-68.2)*** | 4.66 (2.22-8.84)*** | 4.47**** |
| * Interquartile range (19) | |  |  |  |
| ** Range (26) |  |  |  |  |
| *** Simulated from PBPK model of single dose, 5-95% Confidence Intervals (4) | | | | |
| **** Determined from composite profiles (25) | | |  |  |

**S3 Table**. **Comparison of colorectal ARV tissue concentrations in HPTN069/ACTGA5305 and pharmacokinetic studies.**
