# Supplementary material for: Mass spectrometry imaging of hair identifies daily maraviroc adherence in HPTN 069/ACTG A5305
Source: PLoS One. 2023 Jun 23;18(6):e0287449. doi: 10.1371/journal.pone.0287449 (PMC10289441; doi:10.1371/journal.pone.0287449)
Supplement: S4 Table — (DOCX) [file pone.0287449.s010.docx]

| **Analyte** | **Acquisition Polarity** | **Ion Identity** | **Ion m/z** |
| --- | --- | --- | --- |
| Maraviroc | Positive | [M+H]^+^ | 514.33519 |
| Cholesterol | Positive | [M+H-H_2_O]^+^ | 369.35160 |
| PTCA | Negative | [M-H]^-^ | 198.00386 |

**S4 Table**. **IR-MALDESI MSI analytes targeted in hair strand analysis.**
